# Differential and interactive effects of growing and non-growing season precipitation addition on plant and soil stoichiometry in semi-arid steppe

**DOI:** 10.3389/fpls.2026.1861580

**Published:** 2026-06-26

**Authors:** Shiferaw Tolosa Guda, Jiarui Feng, Jingwei Guo, Renhui Miao, Anqun Chen, Yinzhan Liu

**Affiliations:** International Joint Research Laboratory for Global Change Ecology, Laboratory of Biodiversity Conservation and Ecological Restoration, School of Life Sciences, Henan University, Kaifeng, Henan, China

**Keywords:** nitrogen, phosphorus, precipitation regimes, stoichiometry, typical steppe

## Abstract

**Introduction:**

Ecological stoichiometry, which examines the balance of energy and nutrients in ecological interactions, is notably sensitive to variations in precipitation. Seasonal precipitation regimes and their interactions may regulate nutrient mineralization, nutrient mobility, and plant nutrient uptake differently across seasons, thereby altering plant and soil stoichiometric characteristics. However, most studies have emphasized alterations in rainfall during the growing season, often neglecting the role of precipitation during the non-growing season and potential interactions between seasonal precipitation regimes.

**Methods:**

Using a factorial arrangement of two factors of precipitation additions, we conducted a short-term, two-year field experiment to test the differential and interactive effects of non-growing and growing season precipitation additions on plant and soil stoichiometry in a semi-arid steppe.

**Results:**

The results indicated that (1) growing season precipitation addition primarily altered N and P dynamics in both plants and soils, but its effects on stoichiometric balance were strongly dependent on prior moisture conditions; (2) non-growing season precipitation addition exerted legacy effects that altered the magnitude and direction of plant and soil nutrient balances in the subsequent growing season; and (3) interaction between growing and non-growing season precipitation addition fundamentally altered plant and soil stoichiometry. At the community level, growing-season precipitation addition caused a marginal decrease in plant nitrogen concentration under non-growing-season precipitation addition (−1.7 g kg^-1^), whereas no response was observed in its absence. In soil, the presence of non-growing-season precipitation addition influenced the effects of growing-season precipitation addition on C:N and N:P ratios and inorganic N pools. Thus, our findings highlight the importance of considering interactions between the seasonal precipitation regimes when predicting ecosystem nutrient responses to changing climate conditions.

## 1 Introduction

Shifts in the timing and seasonality of precipitation regimes, key features of global climate change, strongly influence plant growth, physiological processes, and overall ecosystem functioning (Wu et al., 2011; Wilcox et al., 2015; Knapp et al., 2016). Precipitation regulates soil moisture regimes and thereby controls the dynamics and bioavailability of key nutrients, including C, N, and P (Craine et al., 2013; Chen et al., 2016; Liu et al., 2016). The stoichiometry of these nutrients, particularly the C:N, C:P, and N:P ratios, is sensitive to changes in precipitation regimes and plays a central role in determining nutrient use efficiency, nutrient limitations, soil fertility, and plant nutritional status (Batjes, 2014; Zhang et al., 2021). For instance, variations in C:N ratios can affect litter decomposition and nutrient cycling, whereas C:P and N:P ratios are closely associated with plant phosphorus limitation, nutrient regulation, and growth strategies in terrestrial ecosystems (Yuan and Chen, 2015; Wang et al., 2021). Elucidating how plant and soil stoichiometry respond to seasonal precipitation is therefore essential for predicting ecosystem responses to climate change, particularly in semi-arid regions.

Precipitation effect on plant and soil C:N:P stoichiometry has been widely documented, yet remain highly variable across ecosystems. In cold desert annuals, increased precipitation reduces leaf and root C:N ratios as well as leaf C:P ratios, whereas perennial species in Inner Mongolia grasslands show weak or no responses in C:N ratios (Liu et al., 2022; Zhang et al., 2022). Similarly, precipitation addition has been associated with increases in leaf and shoot C:P ratios and variable responses in above- and belowground C and N pools (Luo et al., 2022; Li et al., 2023a). These contrasting patterns suggest that precipitation amount alone may not fully explain variation in ecosystem stoichiometric responses, indicating that precipitation timing and seasonal distribution may also play an important role. Because precipitation occurring in different seasons can differentially influence soil moisture availability, plant growth, microbial activity, and nutrient cycling (Nielsen and Ball, 2015), distinct precipitation regimes are likely to generate varying effects on plant and soil nutrient concentrations and their stoichiometric relationships. However, the extent to which seasonal precipitation regimes and their interactions regulate plant and soil C:N stoichiometry remains unclear.

Seasonal precipitation may exert distinct ecological effects because biological activity, soil processes, and plant growth differ substantially between growing and non-growing seasons (Chimner et al., 2010). In particular, winter precipitation can enhance soil moisture carryover into the growing season, thereby influencing plant productivity and soil nutrient cycling (Wu, 2018; Song et al., 2024), whereas growing-season precipitation exerts more direct effects on plant growth and microbial activity (Li et al., 2023a). Such seasonal precipitation dynamics are particularly pronounced in semi-arid grasslands, where strong climatic seasonality governs ecosystem functioning.

The Inner Mongolian temperate steppe is a key component of the temperate grassland ecosystem of northern China and plays an important role in livestock production and biodiversity conservation (Zhang et al., 2020). This semi-arid grassland is characterized by strong seasonal variability in precipitation, making ecosystem processes highly sensitive to changes in moisture regimes (Cheng et al., 2021; Li et al., 2023b). Although previous studies have shown that altered precipitation can influence plant community composition and soil microbial structure in this region, the effects of seasonal precipitation addition and their interactions on plant and soil C, N, and P stoichiometry remain unclear. Addressing this gap is critical for improving predictions of ecosystem responses to changing precipitation regimes under future climate scenarios.

Therefore, we conducted a two-year field experiment to examine the individual and interactive effects of growing and non-growing-season precipitation additions on plant and soil C, N, and P stoichiometry in semi-arid steppe. We hypothesized that: (1) growing season precipitation addition would alter plant and soil C, N, and P concentrations and stoichiometric ratios by increasing soil moisture availability; (2) non-growing season precipitation addition would modify plant and soil C, N, and P concentrations and stoichiometric ratios through its effects on soil moisture carryover and nutrient availability; and (3) the effects of growing season precipitation addition on plant and soil C:N:P stoichiometry would vary with non-growing season precipitation addition.

## 2 Materials and methods

### 2.1 Description of the study area

This study was conducted at the Restoration Ecology and Research Station in Duolun County (42°20′N, 116°17′E), Inner Mongolia Autonomous Region. It is located at an altitude of 1324 m and has a mid-temperate semi-arid continental climate. The long-term mean annual precipitation in the area is 385.5 mm, with an average temperature of 2.4 °C, and the frost-free period is about 100 days. Meteorological data from Duolun County indicate an increasing trend in precipitation over the last five years up to the study period (**Supplementary Figure 1a**). The mean annual precipitation during the study period was 410.3 mm (350.4 mm rainfall and 59.7 mm snowfall). During the study period, the highest snowfall was observed in November (23.4 mm), and the lowest was recorded in December (0.2 mm) (**Supplementary Figure 1b**). The snow melt time was identified on 16^th^ April. The growing season was defined as 16 April to 15 October, whereas the non-growing season was defined as 16 October to 15 April, based on the regional climatic pattern distinguishing the main rainy growing period from the dry non-growing period (Wang et al., 2024). The soil type is chestnut according to the national soil classification, or Haplic Calcisols in the FAO classification. The vegetation type is typical steppe, and the main dominant plants are: *Artemisia frigida*, *Stipa krylovii*, *Leymus chinensis*, *Agropyron cristatum*, and *Cleistogenes squarrosa.* The station was established in 2001 and has good, long-term fenced grassland.

### 2.2 Experimental treatments and design

The experiment was established in 2020 to investigate the interactive effect of precipitation addition during growing and non-growing seasons on plants and soil stoichiometry. The experiment followed a two-factor precipitation addition. It consisted of two levels of non-growing season precipitation addition (NP0, untreated, NP1, non-growing season precipitation addition) and two levels of growing season precipitation addition (GP0, untreated, GP1, growing season precipitation addition). 50% of the total precipitation was added to the plots after each rainfall or snowfall event by a factor of 1. The ambient precipitation sample plots were not treated and denoted by factor 0. Water was added directly via a shower mechanism following each rainfall. Rainfall amounts were calculated for each plot based on plot size and yearly rainfall intensity. Likewise, snow was collected using plastic sheets and added to the experimental plot immediately after each snowfall event each year. The interaction plot received rainfall addition during the growing season and snow augmentation during the non-growing season. A complete randomized block design (CRBD) was used with four replications (**Supplementary Table 2**). We established 16 circular plots, each with a diameter of 3 m and spaced 3 m apart. Experimental treatments were randomly assigned to plots. Within each plot, a central 2 m × 2 m area was designated as the main sampling area, and a fixed 1 m × 1 m sampling area was used.

### 2.3 Plant species

We selected six focal species naturally occurring in our study area. Two species were perennial forbs: *Ixeris chinensis* subsp. *versicolor* (C_3_, 5.5% relative abundance) and *Allium tenuissimum* (C_3_, 3.0% relative abundance). Two were perennial graminoids: *Cleistogenes squarrosa* (C_4_, 9.9% relative abundance) and *Carex* spp. (C_3_, 36.1% relative abundance). Two were perennial sub-shrubs: *Artemisia frigida* (C_3_, 11.4% relative abundance) and *Lespedeza daurica* (C_3_, 4.2% relative abundance). These species collectively accounted for approximately 73.6% of the relative coverage observed across the experimental plots during the study period (**Supplementary Table 3**).

### 2.4 Plant and soil analysis

Aboveground plant parts were collected during the final sampling year of the experiment, at peak biomass in mid-August 2021, from the net plot area (1 × 1 m^2^) in each plot for analysis of carbon, nitrogen, and phosphorus concentrations mill. Aboveground biomass samples were washed with deionized water to remove surface dust and debris, then dried at 60 °C for 72 h, and finally ground using a ball mill (MM400, Retsch, Germany). Total carbon was analyzed using the sulfuric acid digestion method (Gelman et al., 2012), and the total nitrogen concentration was determined using an elemental analyzer (Vario TOC cube, Hanau, Germany). Total phosphorus contents were determined using the molybdenum blue method after digestion (Watanabe and Olsen, 1965).

Soil samples from 0–10 cm and 10–20 cm depths were collected annually from each experimental plot during peak biomass in mid-August 2020 and 2021. The samples were air-dried, ground, and sieved through a 2 mm mesh sieve before laboratory analysis of organic carbon, total nitrogen, available nitrogen, total phosphorus, available phosphorus, and soil pH, using standard laboratory procedures for each parameter.

Soil pH was measured at a 1:2.5 soil liquid ratio (water and 1 *M* KCl solution) (Starter 3100, USA). Soil total organic C (Soil TOC) and soil total N (Soil TN) were determined by the combustion method using the Vario Macro Cube element analyzer (Elementar, Germany). Soil available phosphorus (Soil AP) and Soil total P (Soil TP) were quantified by a colorimetric method using a UV 1900i spectrophotometer (Shimadzu, Kyoto, Japan). Soil nitrate (NO_3_^−^) and ammonium (NH_4_^+^) were determined by the Smart Chem 200 Discrete Auto Analyzer (SYSTEA, Italy) (Norman and Stucki, 1981). All laboratory analyses followed standardized procedures described in the Laboratory Guide for Conducting Soil Tests and Plant Analysis (Jones, 2001).

Plant and soil stoichiometry was calculated as a mass ratio. Soil C:N, C:P, and N:P were determined as the ratio of SOC to TN, SOC to TP, and TN to TP, respectively. We compute the carbon (C), nitrogen (N), and phosphorus (P) concentrations of the plant community and their stoichiometric ratios by averaging the values from selected plant species.

### 2.5 Statistical analysis

A linear mixed-effects model (restricted maximum-likelihood estimation) was used to test the effects of non-growing-season precipitation addition, growing-season precipitation addition, and their interactive effects on the stoichiometry of carbon, nitrogen, and phosphorus in plants and soils. For soil analyses, precipitation addition, year, and their interactions were treated as fixed effects, while block was included as a random effect. Analyses and graphical plots were conducted using R 4.3.2 (R Development Core Team, 2023). Tukey-adjusted emmeans were used in the mixed model fitted with the *lme* function to test *post hoc* pairwise comparisons among treatments. Before analysis, all data were tested for normality and homogeneity of variance.

## 3 Results

### 3.1 Plant C, N, and P stoichiometry

**Figure 1:**
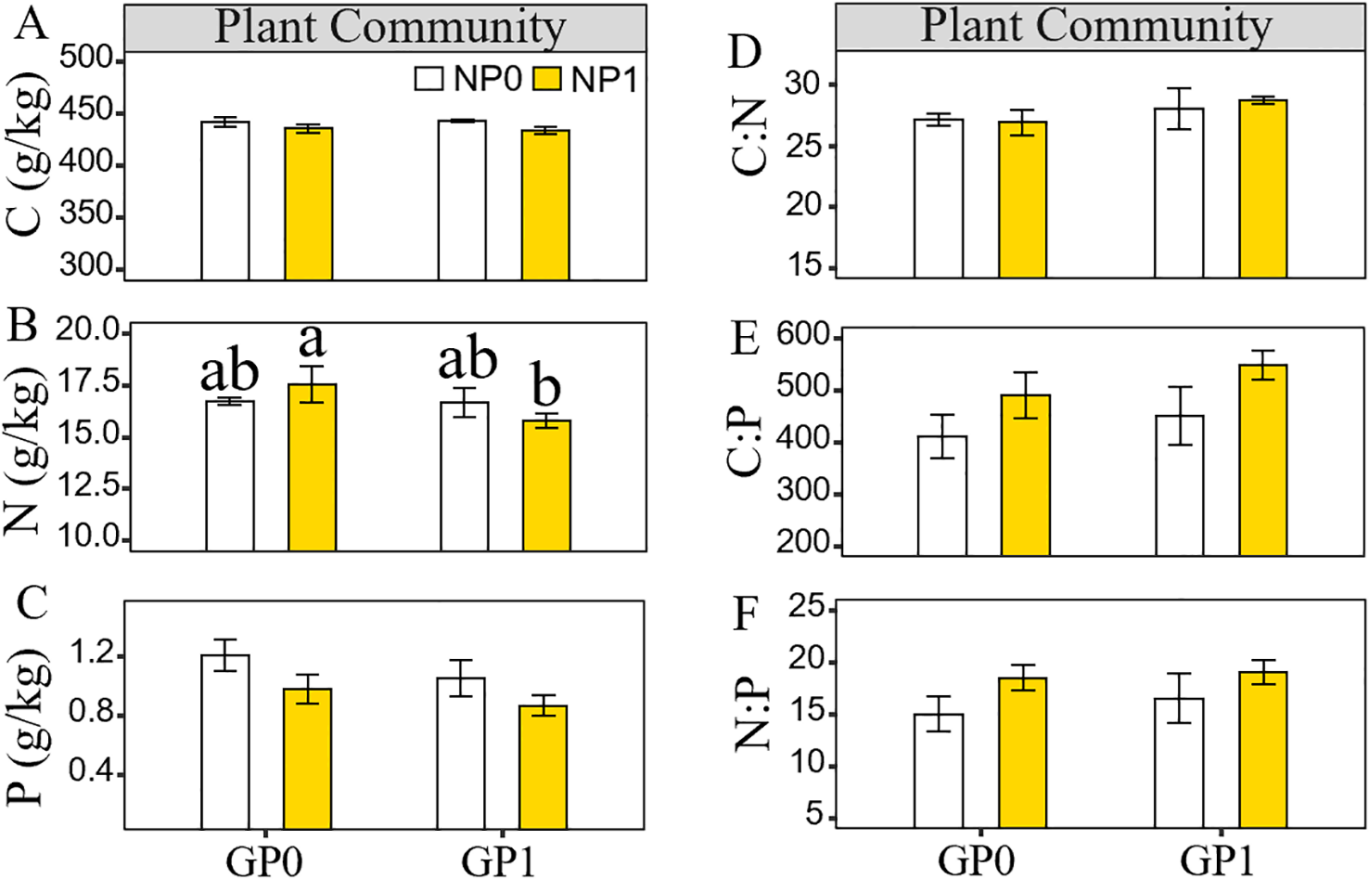
Interactive effect of precipitation addition during non-growing (NP0, NP1) and growing (GP0, GP1) seasons on C, N, and P concentrations and stoichiometric ratios (C:N, C:P, N:P) in a plant community. Bar shows mean values ± SE. Letters above bars indicate significant differences among treatments, as determined by a linear mixed model with two-way ANOVA (*p* < 0.05). 1 indicates the addition of precipitation, while 0 indicates the untreated plot.

Non-growing season precipitation addition (NP) slightly decreased plant community carbon (C) concentration (g kg^-1^) by 7 and markedly reduced phosphorus (P) concentration by 0.207, resulting in higher C:P and N:P ratios by 89 and 3, respectively. NP had no significant effect on nitrogen (N) concentration or the C:N ratio. Growing season precipitation addition (GP) reduced N (g kg^-1^) by 0.9 and P by 0.135, with no effect on C (**Supplementary Table 1**; **Supplementary Figure 3**). No significant interaction between NP and GP was observed for C and P concentrations or stoichiometric ratios; however, under NP, GP resulted in a marginal decrease in N concentration (-1.7), whereas no change was observed in the absence of NP (0 g kg-1) (**Supplementary Table 1**; **Figure 1**).

**Figure 2:**
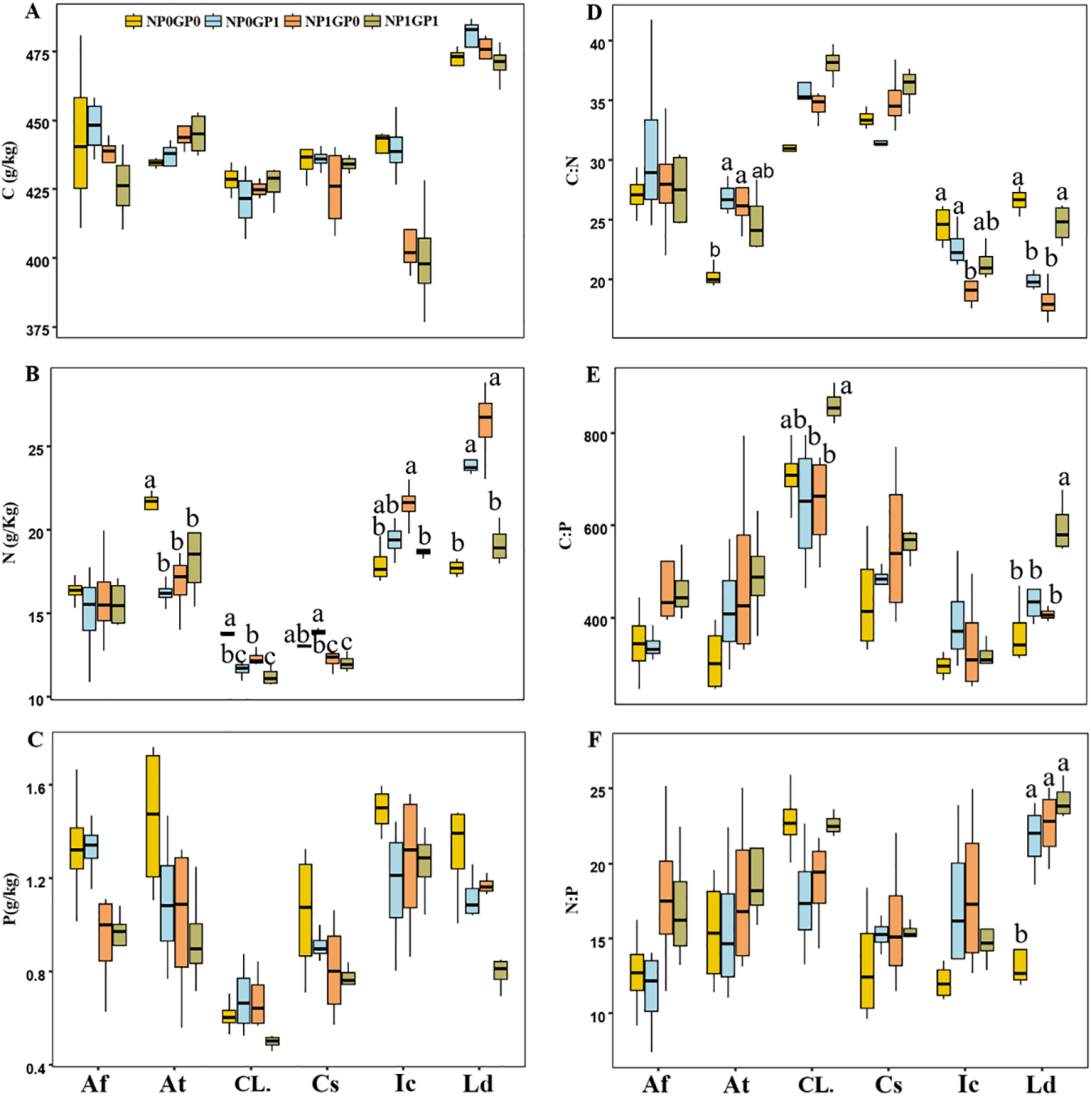
Interactive effect of precipitation addition during non-growing (NP0, NP1) and growing (GP0, GP1) season on plant C, N, and P contents and stoichiometric ratios (C:N, C:P, N:P) in six selected species, Af, *Artemisia frigida*, At, *Allium tenuissimum*, CL., *Carex spp*, Cs, *Cleistogenes squarrosa*, Ic, *Ixeris chinensis* subsp. *Versicolor*, Ld, *Lespedeza daurica* **(A–F)**. Boxplots show the distribution of values for each plant species under each treatment combination (NP0GP0, Control, NP1GP0, non-growing season, NP0GP1, growing season, NP1GP1, annual precipitation addition), with medians, interquartile ranges, and whiskers (1.5× IQR). Letters above boxes indicate significant differences among treatments, as determined by a linear mixed model with two-way ANOVA (*p* < 0.05). 1 indicates the addition of precipitation, while 0 indicates an untreated plot.

At the species level, NP caused species-specific changes in C concentration (g kg^-1^), increasing it in *Allium tenuissimum* (+11) and decreasing it in *Ixeris chinensis* subsp. *versicolor* (-36; **Supplementary Figure 4A**). However, C concentrations remained largely stable under GP (*p* > 0.05, **Supplementary Table 1**). Furthermore, NP and GP did not interact to affect carbon concentration in any of the examined plant species (**Supplementary Table 1**; **Figure 2**). Nitrogen (N) was highly responsive to seasonal precipitation in most examined plants. NP increased N concentration (g kg^-1^) in *Ixeris chinensis* subsp. *versicolor* (+1.7) and *Lespedeza daurica* (+1.9), but decreased it in *Cleistogenes squarrosa* (-0.9) and *Carex* spp. (-1.3), while GP reduced N in *Carex* spp. (-1.7) and *Allium tenuissimum* (-1.9; **Supplementary Figure 4A**).

The interaction of NP × GP was detected for N concentration across species. Under NP, GP reduced N concentration in *Cleistogenes squarrosa* (-1.1), whereas it increased it without NP (+2.2). In *Carex* spp., GP slightly reduced N under NP (-0.2) and increased it without NP (+0.8). In *Allium tenuissimum*, GP increased N under both NP conditions, with a smaller increase under NP (+1.3) than without NP (+5.3). In *Ixeris chinensis* subsp. *versicolor*, GP reduced N under NP (-2.8) but increased it without NP (+1.4), whereas in *Lespedeza daurica*, a strong reduction was observed under NP (-7.2) and an increase without NP (+6.3).

Phosphorus responses varied among species. NP reduced P concentration (g kg^-1^) in *Artemisia frigida* (-0.39) and *Lespedeza daurica* (-0.237), while GP decreased P in *Lespedeza daurica* (-0.289; **Supplementary Figure 4A**), with no significant interactions observed for P in any species (**Supplementary Table 1**; **Figure 2**). Among plant species, C, N, and C:N ratios in *Artemisia frigida* were not significantly affected by moisture increases in any season.

Seasonal precipitation addition led to shifts in stoichiometric ratios, reflecting changes in nutrient balance among species. NP increased C:N in *Cleistogenes squarrosa* (+2.7) and *Carex* spp. (+3.1) but decreased it in *Ixeris chinensis* subsp. *versicolor* (-3.4) and *Lespedeza daurica* (-1.8). NP increased C:P in *Artemisia frigida* (+135) and *Lespedeza daurica* (+104), while N:P increased in *Artemisia frigida* (+5.4) and *Lespedeza daurica* (+5.7). GP increased C:N in *Cleistogenes squarrosa* (+4.4), and increased C:P (+127) and N:P (+4.7) in *Lespedeza daurica* (**Supplementary Figure 4B**). The interaction between NP and GP was observed for C:N, C:P and N:P ratios in certain species. In the presence of NP, GP increased C:N in *Ixeris chinensis* subsp. *versicolor* (+2.5), whereas in the absence of NP it only slightly increased it (+1.8). In *Allium tenuissimum*, GP reduced C:N under NP (-2.1), whereas it increased it in the absence of NP (+0.6). In *Lespedeza daurica*, GP increased C:N under NP (+6.4) but reduced it in the absence of NP (-6.7). Similarly, GP increased C:P in *Cleistogenes squarrosa* under NP (+214) but reduced it in its absence (-66), resulting in a positive interaction effect (+280). Under NP, GP also markedly increased C:P (+189) in *Lespedeza daurica*, whereas only a slight increase was observed in its absence (+63). For N:P ratio, GP increased the ratio in *Cleistogenes squarrosa* under NP (+3.9) but reduced it in its absence (-5.1). In contrast, GP reduced N:P in *Allium tenuissimum* under NP (-3) but increased it in its absence (+5.4). In *Lespedeza daurica*, GP slightly increased N:P under NP (+1.6) and markedly increased it in its absence (+7.9) (**Supplementary Table 1**; **Figure 2**).

### 3.2 Soil C, N, and P dynamics

**Figure 3:**
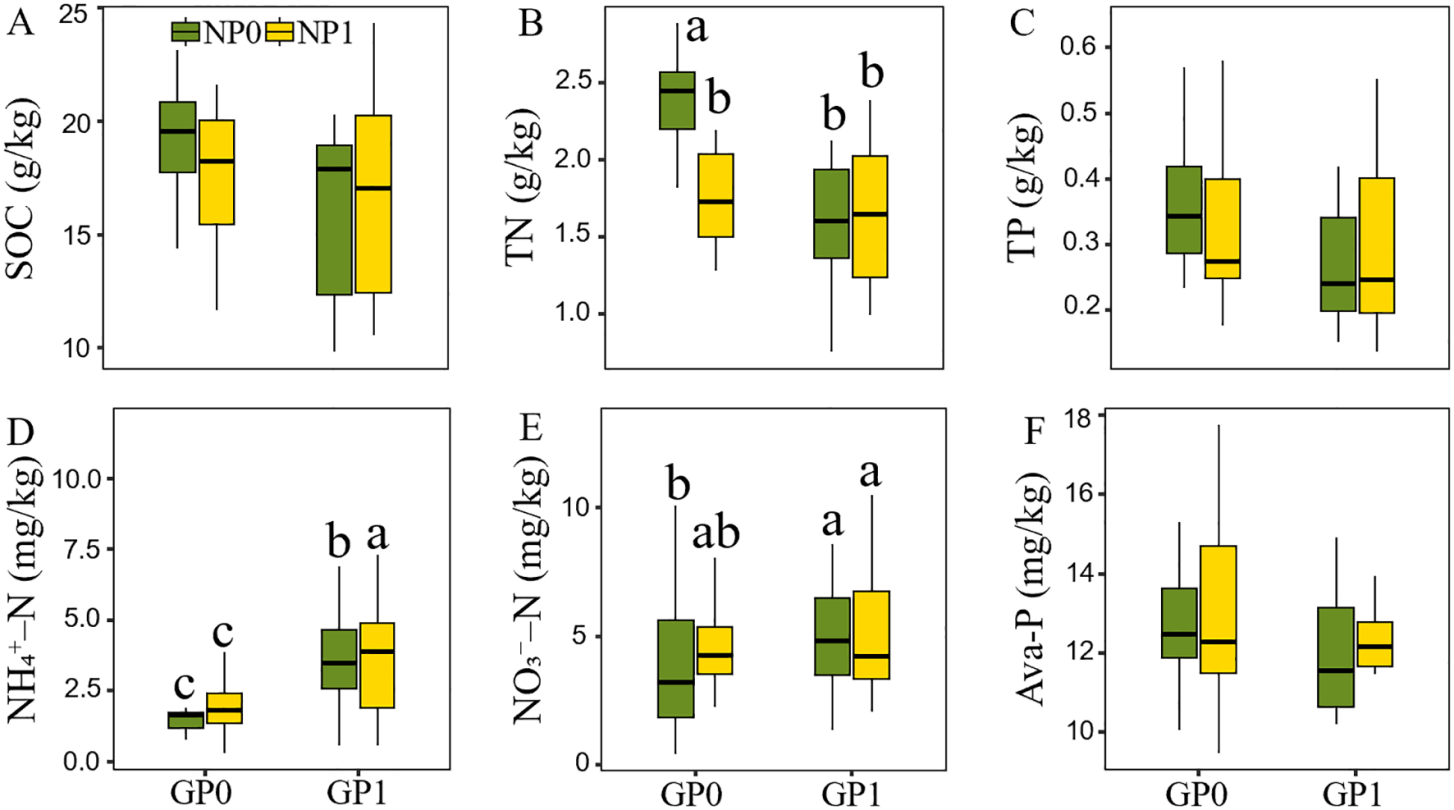
Interactive effect of precipitation addition during non-growing (NP0, NP1) and growing (GP0, GP1) seasons on **(A)** SOC, soil Organic Carbon, **(B)** TN, Total Nitrogen, **(C)** TP, Total Phosphorus, **(D)** NH₄⁺–N, Ammonium, **(E)** NO₃⁻–N, Nitrate, and **(F)** Ava-P, Available Phosphorus. Model details are provided in **Supplementary Table 2**.

The effects of precipitation additions on C, N, and P dynamics were depth-dependent, with some nutrient pools also exhibiting year-specific responses. NP lowered soil organic carbon (SOC) (-1.6) and GP (-4 g kg^-1^) at 10–20 cm but remained unchanged at 0–10 cm (**Supplementary Table 2**; **Supplementary Figure 5**), with no interaction effects detected (**Supplementary Table 2**; **Figure 3**).

**Figure 4:**
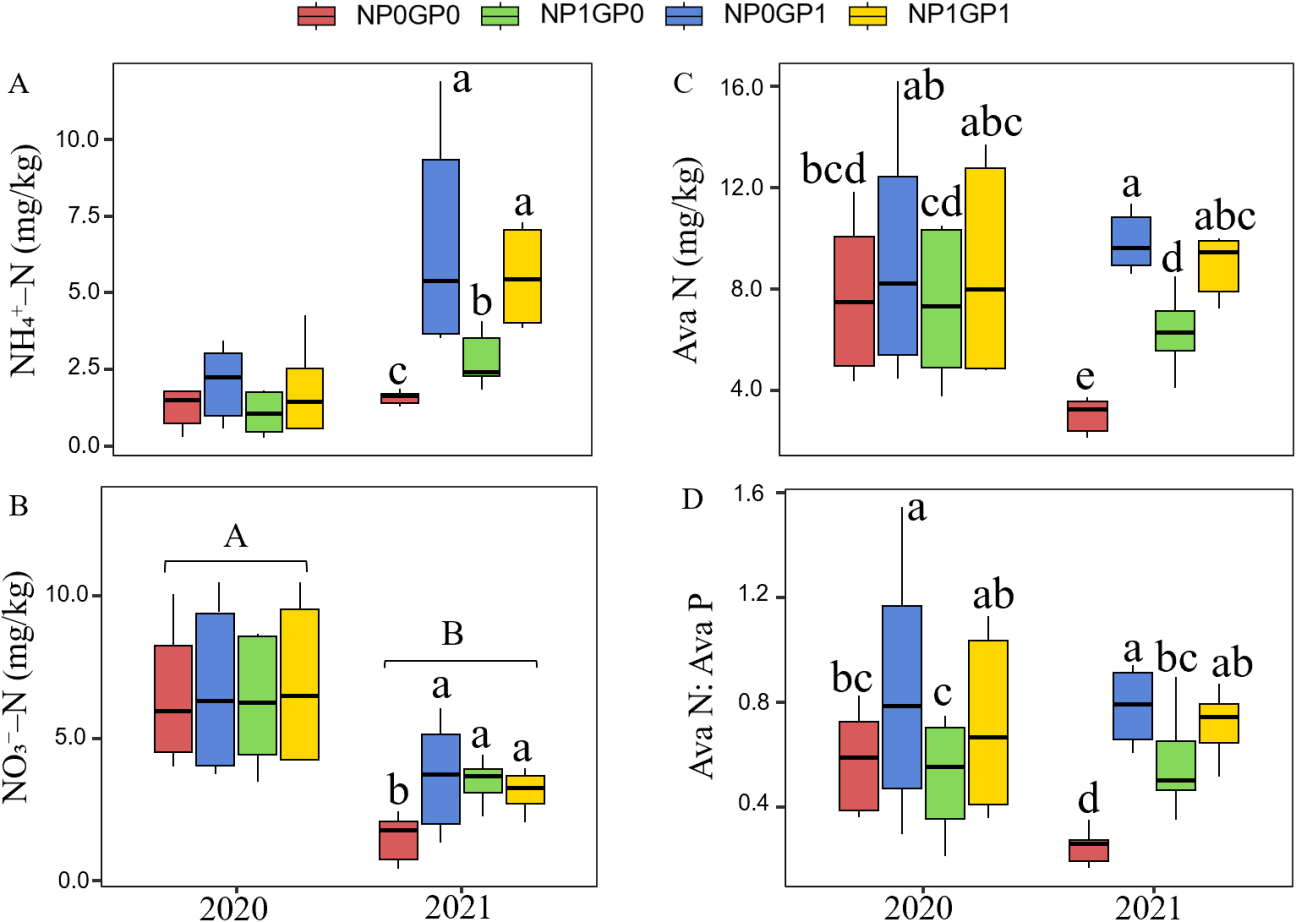
Box plots show the interactive effects of precipitation addition during non-growing and growing seasons, and year on **(A)** Ammonium (NH₄⁺–N), **(B)** Nitrate (NO₃⁻–N), **(C)** available N, and **(D)** Ava N:Ava P, the ratio of available N to available P. In plot B, capital letters (A and B) indicate significant differences among years, whereas lowercase letters indicate significant differences among treatments. 1 indicates the addition of precipitation, and 0 indicates untreated plots.

**Figure 5:**
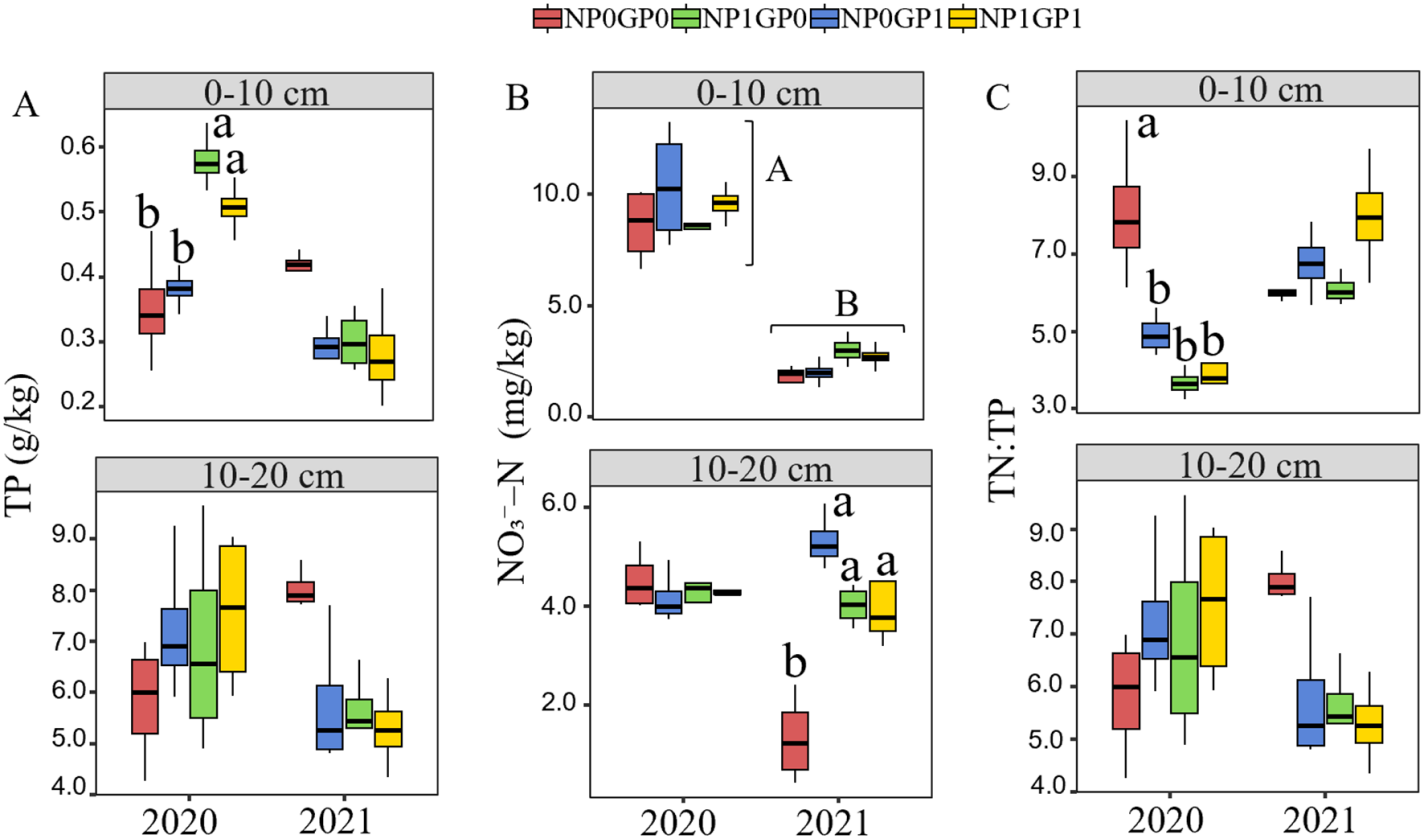
The interactive effects of precipitation addition during non-growing and growing seasons, and year on **(A)** Total Phosphorus (TP), **(B)** Nitrate (NO₃⁻–N), and **(C)** the ratio of TN to TP (TN:TP) at soil samples of 0-10 cm and 10-20 cm. In plot B, capital letters (A and B) indicate significant differences among years, whereas lowercase letters indicate significant differences among treatments. 1 indicates the addition of precipitation, and 0 indicates untreated.

Nitrogen was highly responsive to precipitation treatments. NP reduced total N (TN;-0.22 at 0–10 cm; -0.34 at 10–20 cm) and increased NH_4_^+^–N at 10–20 cm (+1.06 in 2021). GP further reduced TN (-0.29 at 0–10 cm; -0.61 at 10–20 cm) and markedly increased inorganic N pools, including NH_4_^+^–N (+1.28 at 0–10 cm in 2020; +6.37 at 0–10 cm and +1.34 mg kg^-1^at 10–20 cm in 2021) and NO_3_^-^–N (+2.09 mg kg^-1^ at 10–20 cm in 2021; **Supplementary Figure 6**). Under the combined application of NP and GP, TN decreased (-0.1), with a stronger reduction observed in the absence of NP (-0.8; **Supplementary Table 2**; **Figure 3**). In contrast, GP slightly increased NH_4_^+^–N under NP (+2.7), and this increase was greater in the absence of NP (+5; in 2021; **Supplementary Table 2**; **Figure 4**). Similarly, GP increased NO_3_^-^–N under NP (+0.21), whereas a marked increase was observed in the absence of NP (+3.99 at 10–20 cm in 2021; **Supplementary Table 2**; **Figure 5**).

Total phosphorus (TP) also exhibited contrasting responses. NP increased TP (g kg^-1^) at 0–10 cm +0.18 but decreased it at 10–20 cm -0.084, while GP reduced TP at both depths (-0.075 at 0–10 cm in 2021; -0.145 at 10–20 cm in 2020; **Supplementary Figure 5**). Under NP, GP slightly reduced TP at 0–10 cm in 2021 (-0.02), whereas in the absence of NP it markedly reduced TP (-0.129), indicating a positive interaction effect (+0.109) (at 0–10 cm in 2021; **Figure 5**). Available P (mg kg^-1^) was largely unaffected by NP or the interaction, although GP reduced it at 0–10 cm (-1.6) (**Supplementary Table 2**; **Supplementary Figure 5**; **Figure 3**).

**Figure 6:**
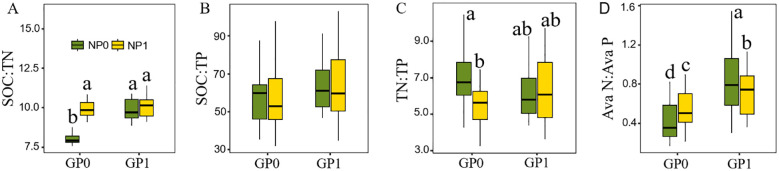
Box plots show the interactive effect of precipitation addition during non-growing and growing seasons on **(A)** SOC:TN, soil organic carbon to total nitrogen, **(B)** SOC:TP, soil organic carbon to total phosphorus, **(C)** TN:TP, total nitrogen to total phosphorus ratios, and **(D)** Ava N: Ava P, available nitrogen to available phosphorus.

Soil stoichiometric ratios further reflected these nutrient dynamics across depths and years. NP increased C:N (+0.86) and affected C:P differently between years (-20 in 2020; +15.6 in 2021; **Supplementary Figure 6**). GP increased C:N (+1.22; **Supplementary Figure 5**) and increased C:P (+16.9 at 0–10 cm in 2021; **Supplementary Figure 6**). In the presence of NP, GP increased the C:N (+0.2), whereas a larger increase was observed in the absence of NP.

(+2.16) No significant effect was observed for C:P at either depth (**Supplementary Table 2**; **Figure 6**).

NP reduced TN:TP (-2.64 at 0–10 cm in 2020), with no effect on the available N:P ratio at either depth. GP decreased the TN:TP ratio (-1.38 at 0–10 cm in 2020) but increased it (+1.34 in 2021; **Supplementary Figure 6**). GP increased the available N:P ratio (+0.49 at 0–10 cm) and (+0.11 at 10–20 cm; **Supplementary Figure 5**). In the presence of NP, GP increased TN:TP (+0.38), whereas it reduced it in the absence of NP (-3.12; 0–10 cm in 2020; **Figure 5**). In 2021, GP reduced TN:TP under NP (-0.41) and more strongly in the absence of NP (-2.27). Moreover, under NP, GP slightly increased the available N:P ratio (+0.151), while a stronger increase was observed in the absence of NP (+0.581), indicating a negative interaction effect (-0.43; p < 0.001; **Supplementary Table 2**; **Figure 4**).

### 3.3 Relationships between soil moisture and plant–soil stoichiometry

**Figure 7:**
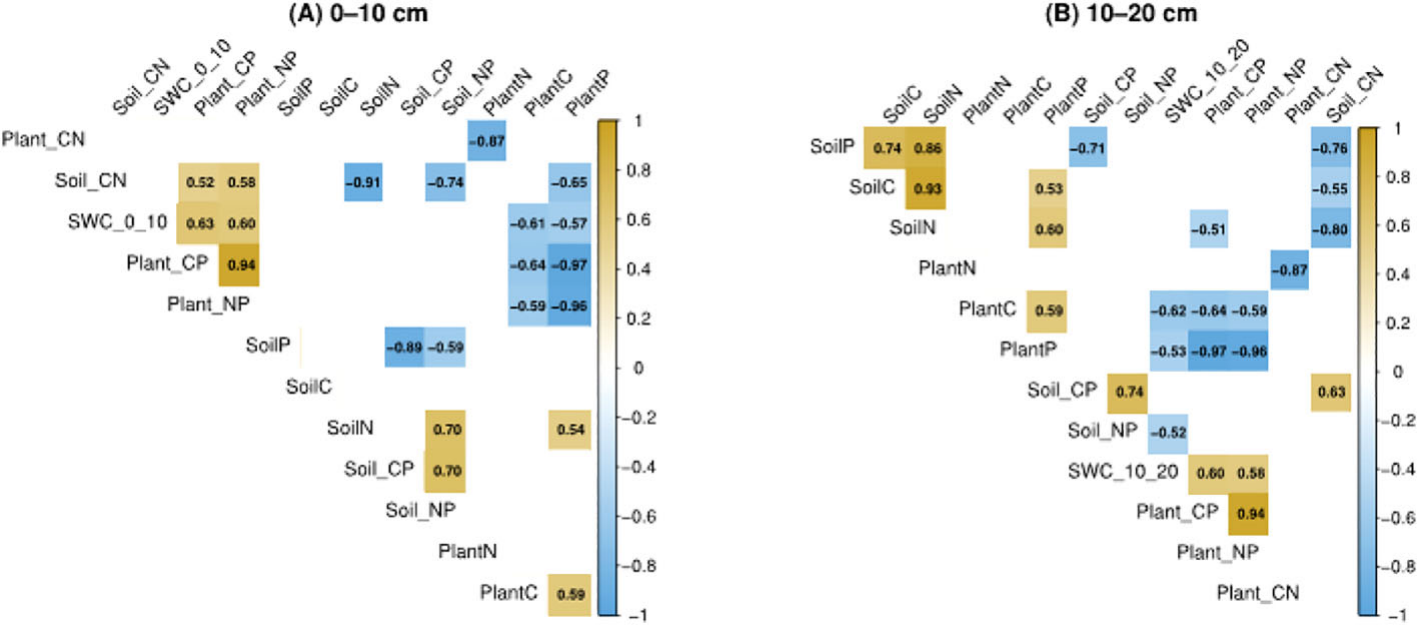
Pearson correlation heatmaps showing relationships between soil moisture and plant and soil C, N, and P stoichiometric variables in a semi-arid grassland. **(A)** 0–10 cm soil layer and **(B)** 10–20 cm soil layer. Variables include plant and soil C, N, and P concentrations and their stoichiometric ratios (C:N, C:P, and N:P). Positive correlations are represented in gold, whereas negative correlations are represented in light blue. Only statistically significant correlations (*p* < 0.05) are displayed, whereas non-significant correlations are left blank. Variables were hierarchically clustered based on similarity in their correlation patterns.

Pearson correlation analyses revealed distinct relationships between soil moisture and plant–soil stoichiometric variables across soil layers (**Figure 7**). In the 0–10 cm soil layer, soil moisture was negatively correlated with plant C concentration (r = -0.61) and plant P concentration (r = -0.57), whereas strong positive correlations were observed with plant C:P (r = 0.63) and N:P ratios (r = 0.60). Correlations between soil moisture and soil nutrient variables were comparatively weaker. In the 10–20 cm soil layer, soil moisture was negatively correlated with plant C concentration (r = -0.62) and plant P concentration (r = -0.53), but positively correlated with plant C:P ratio (r = 0.60). Overall, the 0–10 cm soil layer exhibited stronger relationships between soil moisture and plant stoichiometric characteristics than the 10–20 cm soil layer.

### 3.4 Soil–plant stoichiometric relationships

Relations between soil nutrients and plant community nutrient concentrations clearly showed a significant correlation between plant community and soil stoichiometry. NH4-N, available N, and the ratio of available N to available P were positively correlated with the community C:N ratio. Additionally, community C concentration, community C:N, and the community N:P were positively correlated with the soil TN:TP ratios. However, community N concentration did not significantly correlate with soil stoichiometric traits. Conversely, community P concentration was negatively correlated with available nitrogen, NO_3_^-^-N, and SOC:TN and positively correlated with TN and the TN:TP ratios (**Supplementary Figure 7**).

## 4 Discussion

### 4.1 Main effects of treatments on plant and soil stoichiometry

Precipitation additions during the non-growing (NP) and growing seasons (GP) differentially affected plant and soil stoichiometry. At the plant community level, NP precipitation addition slightly reduced plant carbon (C) and markedly decreased phosphorus (P), whereas GP precipitation addition reduced both N and P, with no effect on C concentration. The relatively stable C concentration suggests that plant carbon assimilation was less sensitive to precipitation variation, whereas N and P responded more strongly to moisture-driven changes in soil nutrient availability, mineralization, and plant uptake processes (Li et al., 2023a). These nutrient shifts subsequently altered community stoichiometry. NP precipitation addition increased C:P and N:P ratios, whereas GP precipitation addition increased C:N and C:P ratios. Together, these contrasting stoichiometric responses indicate that seasonal precipitation regulates community nutrient balance primarily through changes in nitrogen and phosphorus cycling rather than carbon dynamics (Ye et al., 2022).

At the species level, C responses differed mainly under NP precipitation addition, increasing in *Allium tenuissimum* but declining in *Ixeris chinensis* subsp*. versicolor*, while remaining stable under GP precipitation addition, consistent with limited effects of growing-season precipitation on plant C concentration (Li et al., 2016; Wei et al., 2021). N and P responses reflected species-specific differences in nutrient concentrations under seasonal precipitation addition. Under NP precipitation addition, N increased in *Ixeris chinensis* subsp*. versicolor* and *Lespedeza daurica* but declined in *Cleistogenes squarrosa* and *Carex* spp., whereas P decreased mainly in *Artemisia frigida* and *Lespedeza daurica*, likely due to moisture-driven shifts in microbial activity and nutrient redistribution under snowmelt pulses (Chimner et al., 2010). Under GP precipitation addition, N declined in *Carex* spp. and *Allium tenuissimum*, while P decreased only in *Lespedeza daurica*. These shifts generated contrasting stoichiometric responses. NP precipitation addition increased C:N in *Cleistogenes squarrosa* and *Carex* spp. but decreased it in *Ixeris chinensis* subsp*. versicolor* and *Lespedeza daurica*, while both NP and GP precipitation addition increased C:P and N:P, particularly in *Artemisia frigida* and *Lespedeza daurica*, indicating stronger relative phosphorus limitation (Malhotra et al., 2018; Wang et al., 2021; Zhang et al., 2021). Lower C:N in *Ixeris chinensis* subsp*. versicolor* and *Lespedeza daurica* under NP precipitation addition suggest enhanced nitrogen availability via snowmelt-driven mineralization, whereas elevated C:P and N:P reflect constrained phosphorus availability and altered plant nutrient allocation resulting from disproportionate N and P responses to seasonal precipitation addition (Dong et al., 2021; Luo et al., 2022; Rixen et al., 2022).

At the soil level, seasonal precipitation effects were depth- and year-dependent, with stronger responses in nitrogen cycling than in carbon and phosphorus pools. Both NP and GP precipitation additions reduced soil organic carbon (SOC) at 10–20 cm, while 0–10 cm surface SOC remained unchanged, suggesting enhanced decomposition and carbon redistribution in deeper soils (Drahorad et al., 2013). Notably, nitrogen responded strongly to seasonal precipitation additions. NP precipitation addition reduced total nitrogen (TN) at both depths but increased NH_4_^+^–N in deeper soil, whereas GP precipitation addition further decreased TN while increasing NH_4_^+^–N and NO_3_^-^–N across depths, indicating enhanced microbial N mineralization and transformation processes (Hu et al., 2014; Zhang et al., 2017; Fang et al., 2019). Phosphorus responses were comparatively weaker than those of nitrogen but remained strongly dependent on soil depth. NP precipitation addition increased TP at 0–10 cm but reduced it at 10–20 cm, while GP precipitation addition consistently decreased TP and reduced surface available P, likely reflecting increased P loss and redistribution under increased growing-season moisture (Wang et al., 2023b). These changes were reflected in stoichiometric ratios, where NP precipitation addition increased soil C:N and altered C:P in a depth-dependent manner, while GP precipitation addition increased C:N and C:P in specific layers. Both treatments altered TN:TP and available N:P, whereas GP precipitation consistently increased available N:P at both depths, indicating stronger relative phosphorus limitation under growing-season precipitation addition (Yuan et al., 2017). Thus, our results underscore the differential roles of non-growing-season and growing-season moisture in regulating plant stoichiometric responses.

### 4.2 Interactive effects of treatments on plant and soil stoichiometry

The interactions between non-growing-season and growing-season precipitation exerted stronger effects on nitrogen dynamics and stoichiometric ratios than on carbon and phosphorus concentrations at both plant and soil levels. These effects varied notably among plant species and across soil depths and nutrient variables, indicating that non-growing-season precipitation modifies both the magnitude and direction of growing-season precipitation effects on ecosystem stoichiometry (Sun et al., 2021). At the community level, interaction effects were limited, with only a marginal response in plant nitrogen concentration, suggesting relative stability of community-scale nutrient composition, while nitrogen remains the most sensitive element to seasonal moisture coupling (Li et al., 2023a).

At the species level, strong interaction effects were observed for plant N concentration and stoichiometric ratios. Across species, the direction and magnitude of GP precipitation effects depended strongly on NP precipitation conditions, where GP precipitation decreased plant N under NP precipitation addition but increased it in its absence in *Cleistogenes squarrosa*, *Carex* spp., *Ixeris chinensis* subsp*. versicolor*, and *Lespedeza daurica*. This reversal indicates that NP precipitation modifies plant nutrient responses to subsequent growing-season moisture, likely through effects on soil nutrient availability, microbial activity, and plant nutrient uptake dynamics (Zhao et al., 2022). Similar interaction effects were observed for C:N, C:P, and N:P ratios, with species-specific variation in direction and magnitude. GP precipitation addition increased C:P and N:P in *Cleistogenes squarrosa* and *Lespedeza daurica* under NP precipitation addition but had weaker or opposite effects without NP precipitation addition, indicating that NP precipitation modifies nutrient balance under subsequent precipitation inputs, leading to enhanced phosphorus limitation under combined seasonal precipitation effects (Wei et al., 2021). In *Allium tenuissimum*, *Ixeris chinensis* subsp*. versicolor*, and *Lespedeza daurica*, contrasting shifts in C:N further reflect strong interaction effects on carbon–nitrogen balance (Dong et al., 2021). Species differences likely reflect variation in nutrient acquisition strategies and functional traits, including root distribution and plant architecture (Zhou and Jin, 2014; da Silveira Pontes et al., 2015; Coen and Prusinkiewicz, 2024).

In soil, no interaction effects were detected for soil organic carbon, indicating stability of carbon pools under seasonal precipitation manipulation (Hu et al., 2022). In contrast, nitrogen cycling showed strong sensitivity to the interaction between NP and GP precipitation addition, with the absence of NP precipitation amplifying GP precipitation effects, resulting in larger reductions in total nitrogen and stronger increases in inorganic nitrogen (NH_4_^+^–N and NO_3_^-^–N), driven by moisture regulation of mineralization and nitrification processes (Hu et al., 2014; Abbasi et al., 2020), indicating that NP precipitation helps stabilize soil nitrogen pools. Phosphorus responses were weaker, with total phosphorus decreasing in the absence of NP precipitation, while available phosphorus remained largely unchanged, suggesting stronger control by adsorption and immobilization processes (Yuan et al., 2017; Sun et al., 2020). Soil stoichiometric ratios (C:N, TN:TP, and available N:P) showed stronger shifts under absent NP precipitation, indicating enhanced nitrogen limitation patterns under reduced non-growing season precipitation inputs. Pearson correlations further revealed stronger soil moisture–plant stoichiometric relationships in the 0–10 cm layer than in the 10–20 cm layer, where higher moisture was associated with lower plant C and P but higher C:P and N:P ratios. Strong soil–plant coupling was evident under seasonal precipitation manipulation, where precipitation-induced changes in soil nutrient stoichiometry, particularly available N:P, were closely reflected in plant C:N ratios and overall nutrient balance. Consistent with this pattern, Wang et al. (2023a) reported positive relationships between plant nutrient status and soil nutrient concentrations and stoichiometric ratios. Overall, our findings demonstrate that non-growing-season precipitation interacts with growing-season precipitation to regulate plant and soil stoichiometry, highlighting the important role of non-growing-season precipitation in shaping ecosystem responses to subsequent growing-season precipitation.

## 5 Conclusions

Growing-season precipitation addition primarily altered N and P dynamics in both plants and soils, but its effects on stoichiometric balance were strongly dependent on prior moisture conditions. Non-growing-season precipitation addition exerted legacy effects on plant and soil C, N, and P stoichiometry by modifying the magnitude and direction of growing-season moisture effects. Non-growing-season precipitation addition amplified the effects of growing-season precipitation addition, interactively increased plant community-level N concentration, and strongly modified C:N, C:P, and N:P ratios in certain plants. In soils, the interaction between growing and non-growing-season precipitation additions had negative effects on inorganic N pool, C:N, TN:TP, and available N:P ratios, but positive effects on total N and total P. Thus, the present findings reveal that plant and soil stoichiometry are governed primarily by interactive seasonal precipitation effects rather than by individual seasonal inputs. Non-growing-season precipitation exerts strong legacy effects by altering the magnitude and direction of growing-season precipitation effects, thereby regulating nutrient cycling and reshaping plant and soil stoichiometric balance in semi-arid steppe.

## Data Availability

The original contributions presented in the study are included in the article/**Supplementary Material**. Further inquiries can be directed to the corresponding author.
