# Supplementary material for: Differential and interactive effects of growing and non-growing season precipitation addition on plant and soil stoichiometry in semi-arid steppe
Source: Front Plant Sci. 2026 Jun 26;17:1861580. doi: 10.3389/fpls.2026.1861580 (PMC13350334; doi:10.3389/fpls.2026.1861580)

Supplementary Information

Table S1 Results (P and F values) of two-way ANOVA of the effects of non-growing and growing season precipitation addition on C, N, and P content and their stoichiometric ratios in plant species

			**C**				
**Factor**	Af	Cs	C L.	Ic	At	Ld	COMM
NP	**0.136**	**0.214**	**0.752**	**0.001**	**0.013**	**0.748**	**<0.002**
	2.553	1.730	0.100	22.392	8.430	0.110	16.300
GP	**0.749**	**0.268**	**0.430**	**0.723**	**0.984**	**0.890**	**0.784**
	0.107	1.350	0.67	0.132	0.000	0.020	0.080
NP*GP	**0.420**	**0.387**	**0.430**	**0.653**	**0.821**	**0.157**	**0.417**
	0.697	0.810	0.670	0.213	0.050	2.260	0.710
			**N**				
**Factor**	Af	Cs	C L.	Ic	At	Ld	COMM
NP	**0.920**	**0.0001**	**0.001**	**0.020**	**0.103**	**0.023**	**0.903**
	0.011	35.721	17.688	7.245	3.109	6.812	0.015
GP	**0.451**	**0.146**	**<.0001**	**0.222**	**0.031**	**0.538**	**0.012**
	0.606	2.419	54.481	1.657	6.000	0.402	8.628
NP*GP	**0.647**	**0.046**	**0.027**	**0.002**	**0.001**	**<.0001**	**0.017**
	0.221	4.951	6.325	15.477	17.106	88.673	7.589
			**P**				
**Factor**	Af	Cs	C L.	Ic	At	Ld	COMM
NP	**0.002**	**0.067**	**0.280**	**0.584**	**0.075**	**0.003**	**0.002**
	15.499	4.053	1.280	0.318	3.816	13.608	16.860
GP	**1.000**	**0.379**	**0.348**	**0.183**	**0.188**	**0.001**	**0.021**
	0.000	0.835	0.955	1.997	1.951	20.249	7.108
NP*GP	**0.960**	**0.578**	**0.047**	**0.202**	**0.384**	**0.198**	**0.645**
	0.003	0.327	5.315	1.826	0.818	1.853	0.224
			**C:N**				
**Factor**	Af	Cs	C L.	Ic	At	Ld	COMM
NP	**0.610**	**0.002**	**0.004**	**0.001**	**0.074**	**0.019**	**0.658**
	0.275	15.107	12.948	20.254	3.825	7.308	0.206
GP	**0.501**	**0.635**	**0.0001**	**0.655**	**0.076**	**0.871**	**0.020**
	0.482	0.238	36.247	0.210	3.776	0.028	7.245
NP*GP	**0.380**	**0.076**	**0.240**	**0.018**	**0.003**	**<.0001**	**0.391**
	0.830	3.762	1.532	7.460	13.951	93.486	0.793
			**C:P**				
**Factor**	Af	Cs	C L.	Ic	At	Ld	COMM
NP	**0.013**	**0.093**	**0.149**	**0.719**	**0.092**	**0.002**	**0.002**
	8.559	3.341	2.380	0.136	3.361	16.681	16.418
GP	**0.703**	**0.720**	**0.171**	**0.346**	**0.464**	**0.0003**	**0.044**
	0.152	0.134	2.121	0.963	0.573	24.802	5.079
NP*GP	**0.785**	**0.712**	**0.017**	**0.153**	**0.445**	**0.029**	**0.692**
	0.078	0.143	7.612	2.329	0.624	6.112	0.165
			**N:P**				
**Factor**	Af	Cs	C L.	Ic	At	Ld	COMM
NP	**0.021**	**0.356**	**0.774**	**0.382**	**0.191**	**0.0003**	**0.004**
	7.106	0.923	0.086	0.823	1.918	24.584	12.645
GP	**0.609**	**0.620**	**0.652**	**0.554**	**0.645**	**0.001**	**0.250**
	0.275	0.260	0.214	0.370	0.224	17.284	1.462
NP*GP	**0.927**	**0.439**	**0.008**	**0.053**	**0.726**	**0.018**	**0.575**
	0.009	0.640	10.146	4.600	0.129	7.508	0.332

Notes: F-value and *p*-value are shown. The p-values are bolded. The Numerator df and Denominator df for all factors = (1,12). Af, *Artemisia frigida*, Cs, *Cleistogenes squarrosa*, Cl., *Carex spp*, Ic, *Ixeris chinensis* subsp. *Versicolor*, At, *Allium tenuissimum*, Ld, *Lespedeza daurica* and COMM, community level, Factor (NP, non-growing season precipitation, GP, growing season precipitation)

Table S2 Linear mixed effect model was used to investigate the effect of precipitation addition (non-growing season precipitation, NP and growing season precipitation, GP), year (Y), and soil layer (SL) on soil organic carbon, SOC, total nitrogen, TN, total phosphorus, TP, available phosphorus, Ava P, available nitrogen, Ava N, ammonium, NH4-N, and nitrate, NO3-N concentration, ratios of soil organic carbon to total nitrogen, SOC:TN, soil organic carbon to total phosphorus, SOC:TP, total nitrogen to total phosphorus, TN:TP, available nitrogen to available phosphorus, Av N:Av P during 2020 and 2021 growing and non-growing season

	Factor	numDF	denDF	F-value	p-value
**SOC**	**(Intercept)**	1	36	3589.741	**<.0001**
	**NP**	1	12	0.737	0.4076
	**GP**	1	12	12.606	**0.004**
	**Y**	1	36	14.281	**0.0006**
	**SL**	1	36	159.512	**<.0001**
	**NP:GP**	1	12	4.687	0.0513
	**NP:Y**	1	36	0.486	0.4904
	**GP:Y**	1	36	4.795	**0.0351**
	**NP:SL**	1	36	7.86	**0.0081**
	**GP:SL**	1	36	20.641	**0.0001**
	**Y:SL**	1	36	1.972	0.1688
	**NP:GP: Y**	1	36	0.457	0.5033
	**NP:GP:SL**	1	36	0.093	0.7623
	**NP:Y:SL**	1	36	1.791	0.1892
	**GP:Y:SL**	1	36	0.013	0.9095
	**NP:GP:Y:SL**	1	36	0.506	0.4813
**TN**	**(Intercept)**	1	36	4056.807	**<.0001**
	**NP**	1	12	23.286	**0.0004**
	**GP**	1	12	60.463	**<.0001**
	**Y**	1	36	6.326	**0.0165**
	**SL**	1	36	163.718	**<.0001**
	**NP:GP**	1	12	36.512	**0.0001**
	**NP:Y**	1	36	0.008	0.9285
	**GP:Y**	1	36	4.515	**0.0405**
	**NP:SL**	1	36	1.781	0.1904
	**GP:SL**	1	36	11.868	**0.0015**
	**Y:SL**	1	36	0.246	0.6231
	**NP:GP:Y**	1	36	2.468	0.125
	**NP:GP:SL**	1	36	1.835	0.184
	**NP:Y:SL**	1	36	0.021	0.8843
	**GP:Y:SL**	1	36	2.377	0.1319

	**NP:GP:Y:SL**	1	36	0.607	0.4412
**TP**	**(Intercept)**	1	36	2100.062	**<.0001**
	**NP**	1	12	0.4519	0.5142
	**GP**	1	12	26.9162	**0.0002**
	**Y**	1	36	29.6016	**<.0001**
	**SL**	1	36	107.977	**<.0001**
	**NP:GP**	1	12	2.1908	0.1646
	**NP:Y**	1	36	7.0857	**0.0115**
	**GP:Y**	1	36	0.9966	0.3248
	**NP:SL**	1	36	12.3643	**0.0012**
	**GP:SL**	1	36	2.8182	0.1019
	**Y:SL**	1	36	17.8231	**0.0002**
	**NP:GP:Y**	1	36	1.9006	0.1765
	**NP:GP:SL**	1	36	1.9283	0.1735
	**NP:Y:SL**	1	36	35.1879	**<.0001**
	**GP:Y:SL**	1	36	8.4509	**0.0062**
	**NP:GP:Y:SL**	1	36	6.0224	**0.0191**
**Ava P**	**(Intercept)**	1	36	4752.476	**<.0001**
	**NP**	1	12	0.691	0.4221
	**GP**	1	12	3.577	0.083
	**Y**	1	36	3.803	0.059
	**SL**	1	36	0.098	0.7558
	**NP:GP**	1	12	0.046	0.8331
	**NP:Y**	1	36	3.706	0.0621
	**GP:Y**	1	36	15.39	**0.0004**
	**NP:SL**	1	36	0.478	0.4939
	**GP:SL**	1	36	6.169	**0.0178**
	**Y:SL**	1	36	0.182	0.6726
	**NP:GP:Y**	1	36	0.835	0.367
	**NP:GP:SL**	1	36	3.287	0.0782
	**NP:Y:SL**	1	36	1.064	0.3092
	**GP:Y:SL**	1	36	0.462	0.5011
	**NP:GP:Y:SL**	1	36	0.653	0.4243
**NH4-N**	**(Intercept)**	1	36	1181.101	**<.0001**
	**NP**	1	12	0.0234	0.8809
	**GP**	1	12	197.4622	**<.0001**
	**Y**	1	36	253.2069	**<.0001**
	**SL**	1	36	121.1298	**<.0001**
	**NP:GP**	1	12	13.4595	**0.0032**
	**NP:Y**	1	36	1.2077	0.2791
	**GP:Y**	1	36	93.1215	**<.0001**
	**NP:SL**	1	36	5.8977	**0.0203**
	**GP:SL**	1	36	89.8071	**<.0001**
	**Y:SL**	1	36	0.0775	0.7824
	**NP:GP:Y**	1	36	11.7655	**0.0015**
	**NP:GP:SL**	1	36	1.8814	0.1787

	**NP:Y:SL**	1	36	10.7753	**0.0023**
	**GP:Y:SL**	1	36	38.0942	**<.0001**
	**NP:GP:Y:SL**	1	36	2.0232	0.1635
**NO3-N**	**(Intercept)**	1	36	1147.687	**<.0001**
	**NP**	1	12	1.1849	0.2977
	**GP**	1	12	8.6898	**0.0122**
	**Y**	1	36	250.8084	**<.0001**
	**SL**	1	36	57.9175	**<.0001**
	**NP:GP**	1	12	3.9213	0.0711
	**NP:Y**	1	36	6.1881	**0.0176**
	**GP:Y**	1	36	0.7305	0.3984
	**NP:SL**	1	36	0.0234	0.8793
	**GP:SL**	1	36	0.3606	0.552
	**Y:SL**	1	36	179.7618	**<.0001**
	**NP:GP:Y**	1	36	4.9703	**0.0321**
	**NP:GP:SL**	1	36	1.132	0.2944
	**NP:Y:SL**	1	36	0.3471	0.5594
	**GP:Y:SL**	1	36	14.5948	**0.0005**
	**NP:GP:Y:SL**	1	36	5.2246	**0.0283**
**Ava N**	**(Intercept)**	1	36	1801.398	**<.0001**
	**NP**	1	12	0.6348	0.4411
	**GP**	1	12	75.3741	**<.0001**
	**Y**	1	36	21.1671	**0.0001**
	**SL**	1	36	193.3195	**<.0001**
	**NP:GP**	1	12	10.4295	**0.0072**
	**NP:Y**	1	36	8.8713	**0.0052**
	**GP:Y**	1	36	46.7321	**<.0001**
	**NP:SL**	1	36	2.7662	0.105
	**GP:SL**	1	36	29.032	**<.0001**
	**Y:SL**	1	36	148.0029	**<.0001**
	**NP:GP:Y**	1	36	17.6585	**0.0002**
	**NP:GP:SL**	1	36	0.0142	0.9057
	**NP:Y:SL**	1	36	2.2969	0.1384
	**GP:Y:SL**	1	36	0.1246	0.7261
	**NP:GP:Y:SL**	1	36	1.4692	0.2334
**SOC:TN**	**(Intercept)**	1	36	3500.047	**<.0001**
	**NP**	1	12	7.151	**0.0203**
	**GP**	1	12	13.278	**0.0034**
	**Y**	1	36	0.352	0.5566
	**SL**	1	36	1.12	0.2969
	**NP:GP**	1	12	9.692	**0.009**
	**NP:Y**	1	36	0.014	0.9053
	**GP:Y**	1	36	0.057	0.8127
	**NP:SL**	1	36	0.581	0.451
	**GP:SL**	1	36	0.283	0.5982

	**Y:SL**	1	36	0.101	0.7521
	**NP:GP:Y**	1	36	1.493	0.2296
	**NP:GP:SL**	1	36	0.927	0.3422
	**NP:Y:SL**	1	36	2.75	0.1059
	**GP:Y:SL**	1	36	2.937	0.0952
	**NP:GP:Y:SL**	1	36	0.188	0.6673
**SOC:TP**	**(Intercept)**	1	36	1337.787	**<.0001**
	**NP**	1	12	0.00	0.9976
	**GP**	1	12	4.1967	0.063
	**Y**	1	36	0.7094	0.4052
	**SL**	1	36	6.891	**0.0126**
	**NP:GP**	1	12	0.0144	0.9066
	**NP:Y**	1	36	0.9187	0.3442
	**GP:Y**	1	36	0.096	0.7585
	**NP:SL**	1	36	0.5728	0.4541
	**GP:SL**	1	36	0.0093	0.9236
	**Y:SL**	1	36	19.6421	**0.0001**
	**NP:GP:Y**	1	36	0.5216	0.4748
	**NP:GP:SL**	1	36	0.5978	0.4445
	**NP:Y:SL**	1	36	25.1711	**<.0001**
	**GP:Y:SL**	1	36	10.2879	**0.0028**
	**NP:GP:Y:SL**	1	36	2.3794	0.1317
**TN:TP**	**(Intercept)**	1	36	1951.807	**<.0001**
	**NP**	1	12	5.5401	**0.0365**
	**GP**	1	12	0.0885	0.7712
	**Y**	1	36	2.1249	0.1536
	**SL**	1	36	4.5823	**0.0391**
	**NP:GP**	1	12	6.2841	**0.0276**
	**NP:Y**	1	36	1.102	0.3008
	**GP:Y**	1	36	0.0861	0.7709
	**NP:SL**	1	36	1.3384	0.2549
	**GP:SL**	1	36	0.0509	0.8228
	**Y:SL**	1	36	15.3586	**0.0004**
	**NP:GP:Y**	1	36	0.0083	0.9281
	**NP:GP:SL**	1	36	2.4149	0.1289
	**NP:Y:SL**	1	36	22.9147	**<.0001**
	**GP:Y:SL**	1	36	20.2776	**0.0001**
	**NP:GP:Y:SL**	1	36	4.9993	**0.0316**
**AvN:AvP**	**(Intercept)**	1	36	1414.565	**<.0001**
	**NP**	1	12	0.0146	0.9059
	**GP**	1	12	81.2337	**<.0001**
	**Y**	1	36	6.2984	**0.0167**
	**SL**	1	36	95.1053	**<.0001**
	**NP:GP**	1	12	14.12	**0.0027**
	**NP:Y**	1	36	11.3087	**0.0018**
	**GP:Y**	1	36	4.6363	**0.0381**

**NP:SL**	1	36	6.3547	**0.0163**
**GP:SL**	1	36	40.1412	**<.0001**
**Y:SL**	1	36	67.1475	**<.0001**
**NP:GP:Y**	1	36	8.8354	**0.0052**
**NP:GP:SL**	1	36	1.1783	0.2849
**NP:Y:SL**	1	36	1.9259	0.1737
**GP:Y:SL**	1	36	0.5313	0.4708
**NP:GP:Y:SL**	1	36	1.743	0.1951

Notes: F-value and p-value are shown. The p-values lower than alpha 0.05 are bolded. Factor (NP, non-growing season precipitation, GP, growing season precipitation, Y, Year, SL, Soil layer).

Table S3 The sampled six common species, photosynthetic pathway (PP), life history traits (LH), their relative abundance (%), and relative cover (%) within the quadrats

Species	Abbrev.	LF	PP	R. Abund. (%)	S. Cover (%)
Ixeris chinensis subsp. Versicolor	Ic	Forb	C3	5.5	3.5
Allium tenuissimum	At	Forb	C3	3.0	0.3
Cleistogenes squarrosa	Cs	Grass	C4	9.9	11.6
Carex spp	Cl.	Grass	C3	36.1	5.4
Artemisia frigida	Af	Sub-shrubs	C3	11.4	47.6
Lespedeza daurica	Ld	Sub-shrubs	C3	4.2	5.2

Figure S1 The inter-annual and intra-annual distribution of precipitation. (a) illustrates the inter-annual mean annual precipitation (MAP) from 2016 to 2020. (b) shows the intra-annual mean annual precipitation during the study period

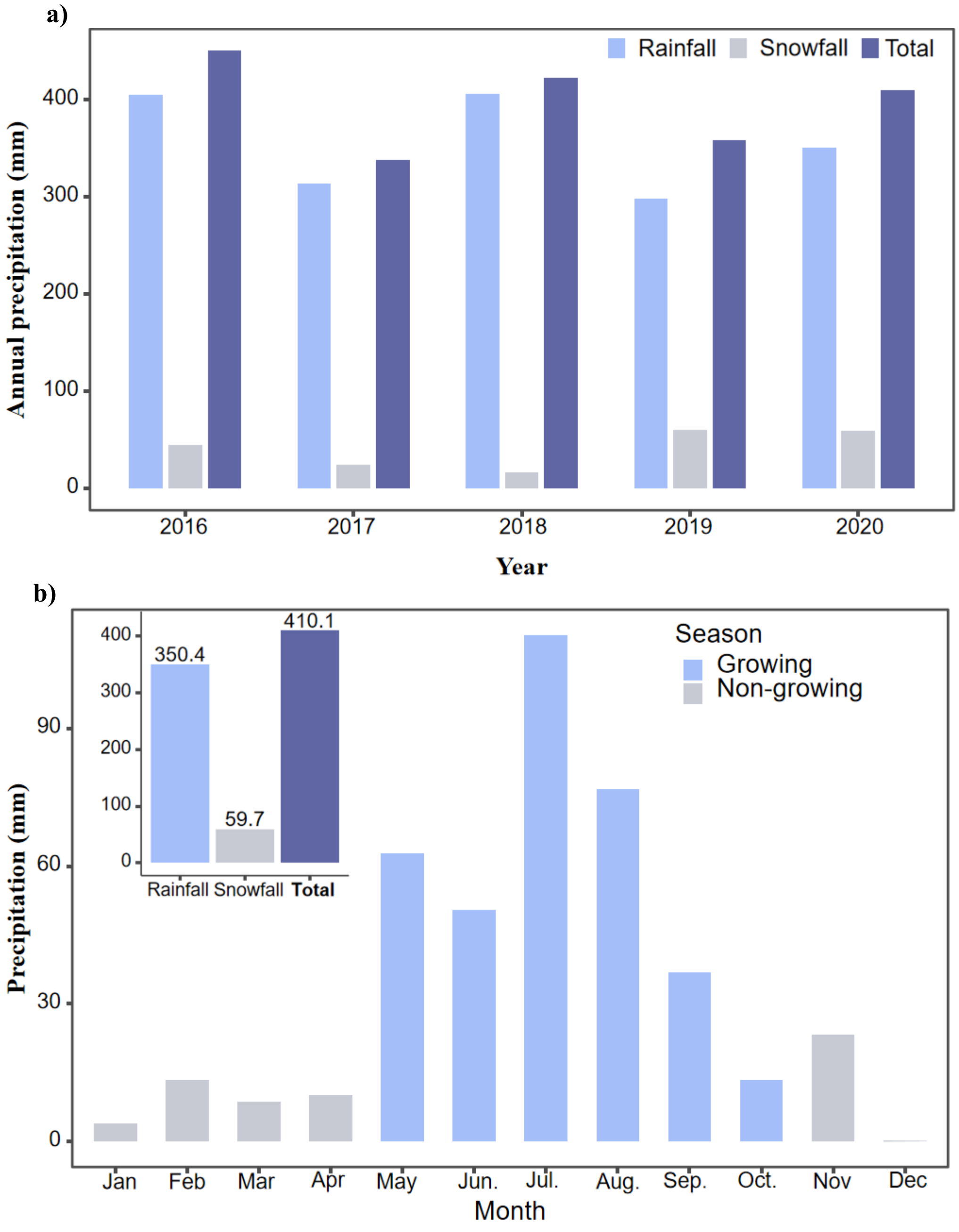

Figure S2 The distribution of the 16 plots under precipitation manipulation experiment during growing and non-rowing seasons (C, control, NP, non-growing season precipitation addition, GP, growing season precipitation addition, AP, Annual precipitation addition)

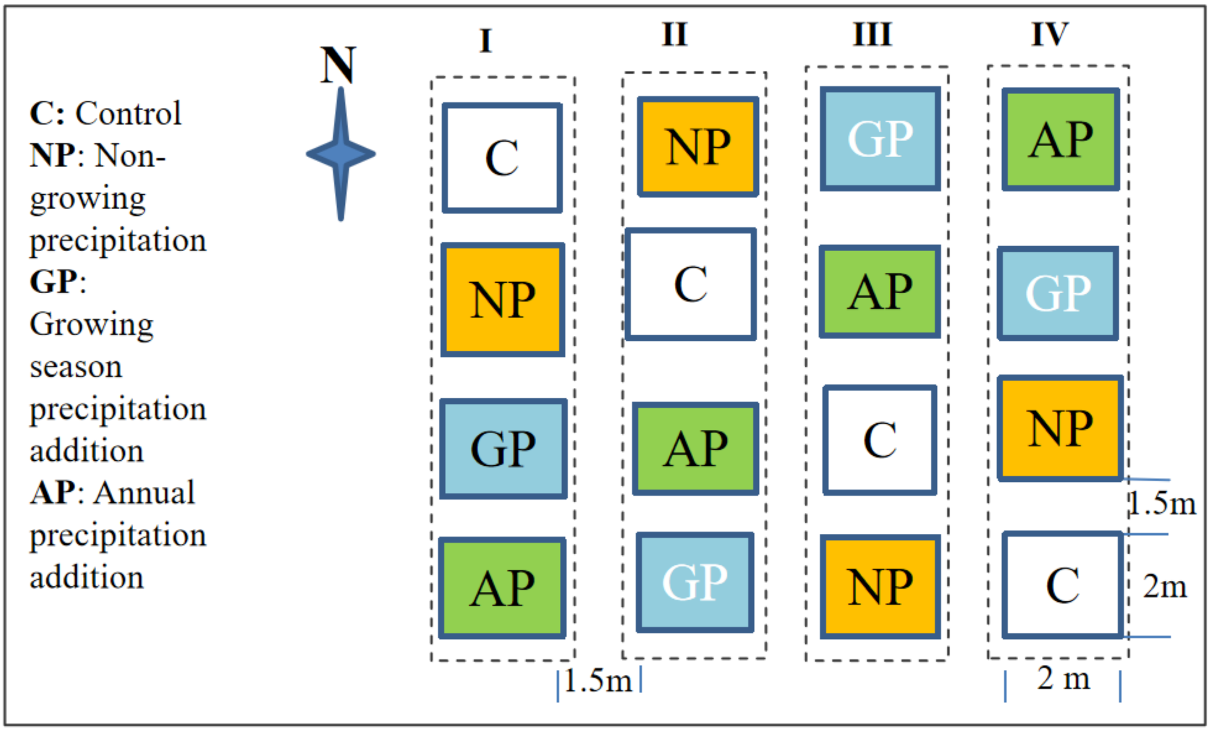

Figure S3 Main effect of growing and non-growing season precipitation on carbon, nitrogen, and phosphorus contents and their stoichiometric ratios of plant community. Mixed lme models were used to test the effect of precipitation addition (growing season precipitation addition, GP) and (Non-growing precipitation addition, NP) on stoichiometry in the typical steppe of Inner Mongolia. EMMEANS Tukey adjustment were used to test pairwise comparisons of differences among treatments. Violin box plots without letters are not significantly different from each other at alpha 0.05 probability level. 1 indicates addition of precipitation while 0 indicates untreated plot

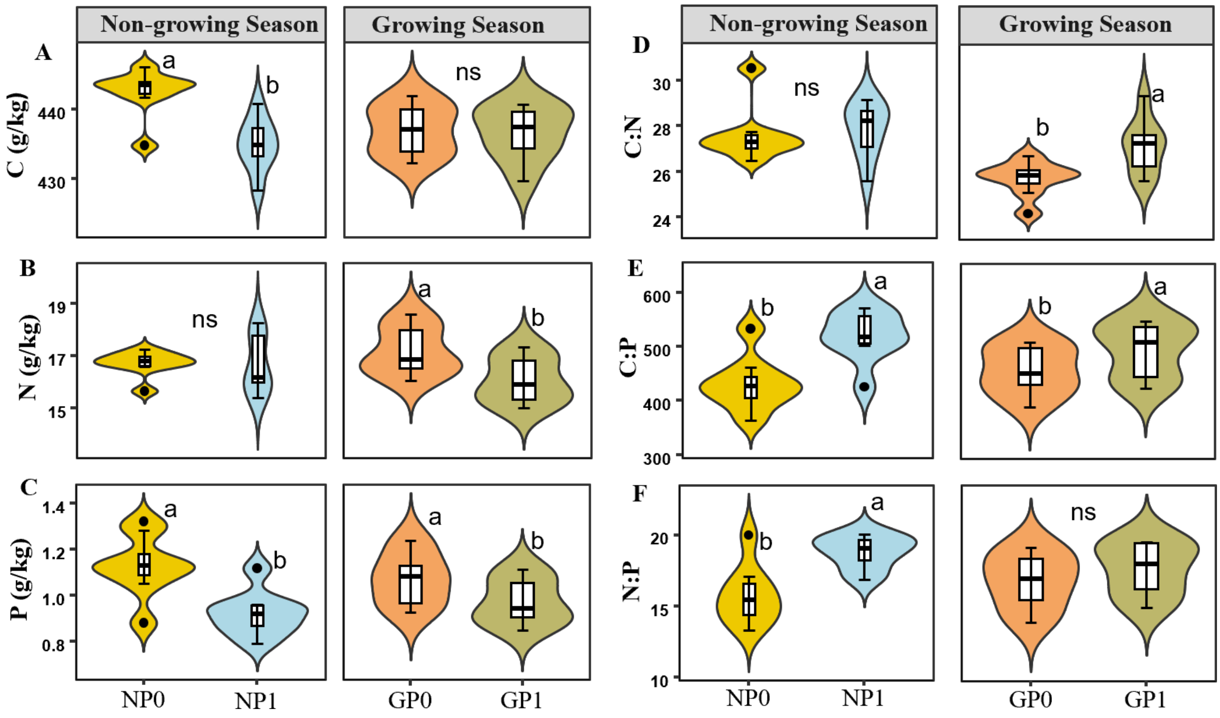

Figure S4 Main effect of growing and non-growing season precipitation addition on carbon, nitrogen, and phosphorus contents and their stoichiometric ratios of six plant species, Af, *Artemisia frigida,* At, *Allium tenuissimum,* CL., *Carex spp,* Cs, *Cleistogenes squarrosa,* Ic, *Ixeris chinensis* subsp. *Versicolor,* Ld, *Lespedeza daurica*. Mixed lme models were used to test the effect of precipitation addition (growing season precipitation addition, GP) and (non-growing season precipitation addition, NP) on stoichiometry in the typical steppe of Inner Mongolia. EMMEANS Tukey adjustment were used to test pairwise comparisons of differences among treatments. A box plot without letters are not significantly different from each other at alpha 0.05 probability level. 1 indicates addition of precipitation while 0 indicates untreated plot

A)

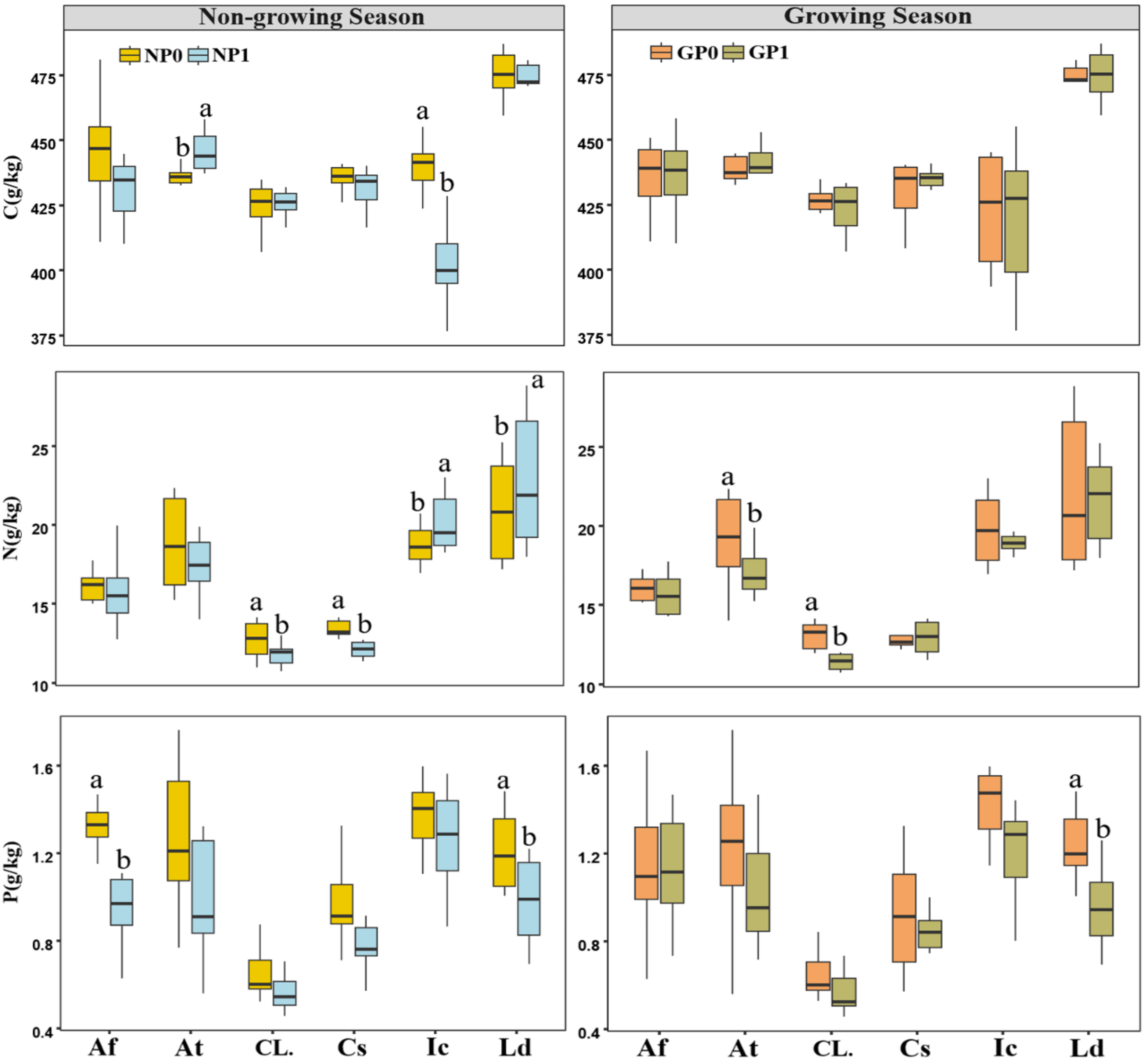

B)

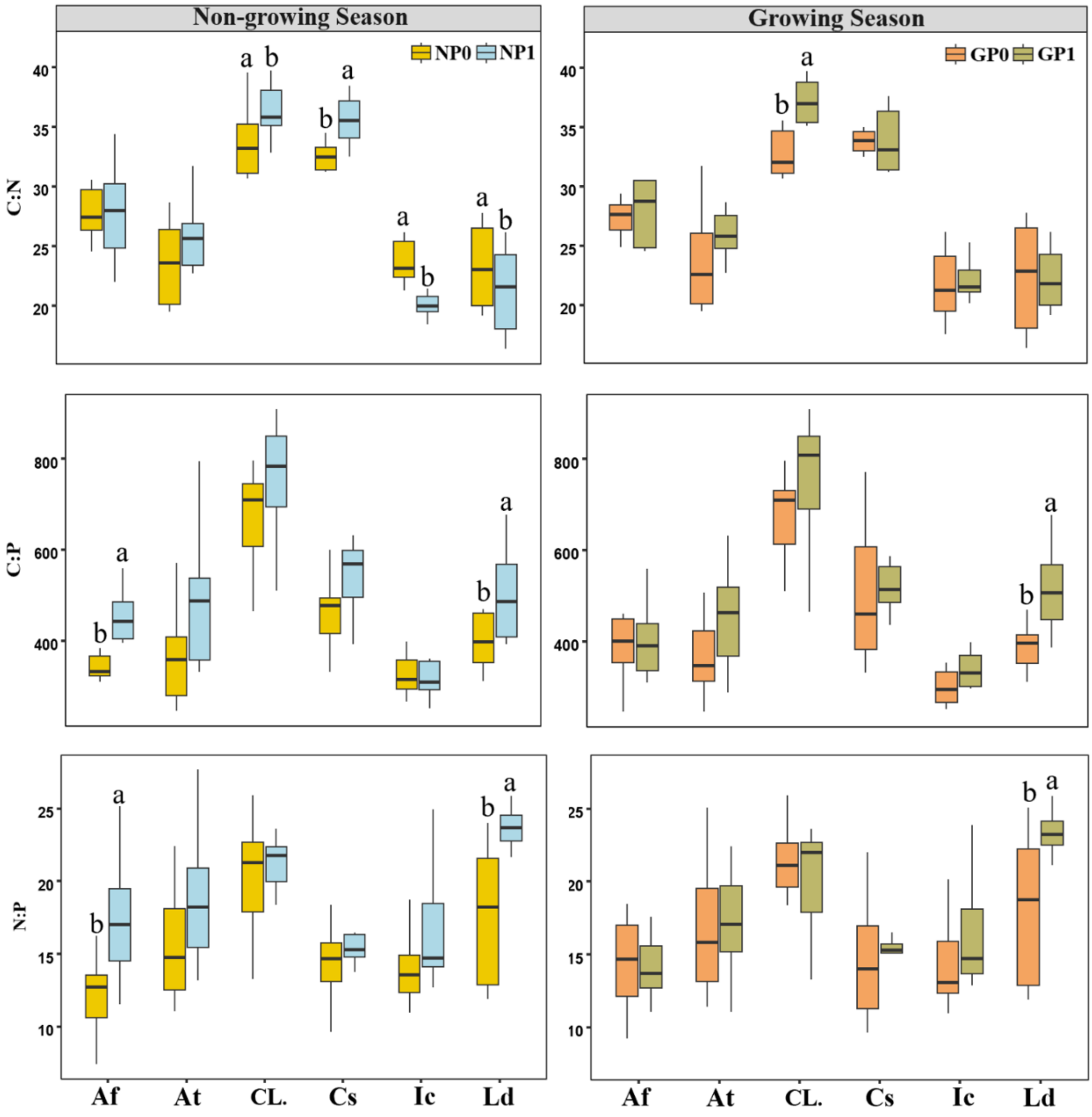
Non-growing Season
Growing Season
NP0
NP1
GP0
GP1
C:N
C:P
N:P
Af
At
CL.
Cs
Ic
Ld

Figure S5 Main effects of growing and non-growing season precipitation addition (NP, non-growing season precipitation addition, GP, growing season precipitation addition) on Soil Organic Carbon (SOC), Total Nitrogen (TN), Total Phosphorus (TP), Available Phosphorus (Ava P), Available Nitrogen (Ava N), Ammonium (NH4-N) and Nitrate (NO3-N) at Soil samples of 0-10 cm (S1) and 10-20 cm (S2). Mixed lme models were used to test the effects of precipitation addition in growing and non-growing seasons on stoichiometry in the typical steppe of Inner Mongolia. EMMEANS Tukey adjustment were used to test post hoc pairwise comparisons of differences among treatments. Error bars with *** indicates highly significant at $p < 0.001$; ** significant at $p < 0.01$; * significant at $p < 0.05$; ns = not significant at $p \geq 0.05$. 1 indicates the addition of precipitation, and 0 indicates untreated

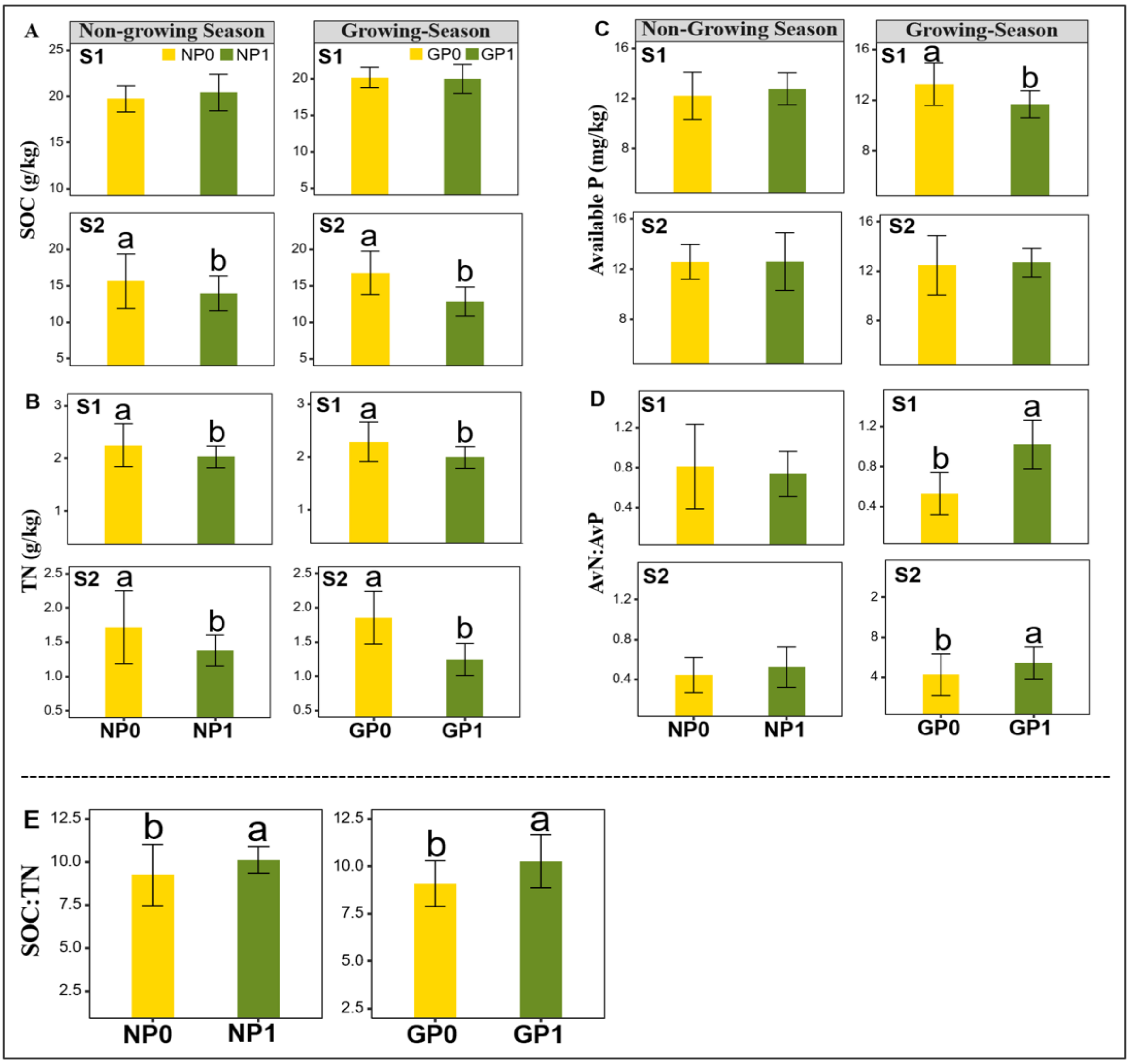

Figure S6 Main effects of growing and non-growing season precipitation addition (NP, non-growing season precipitation addition, GP, growing season precipitation addition) and year on Total Phosphorus (TP), Ammonium (NH4-N), Nitrate (NO3-N), the ratio of soil organic carbon to total phosphorus (C:P) and the ratio of available nitrogen to available phosphorus (Ava N: Ava P) at soil samples of 0-10 cm (S1) and 10-20 cm (S2). Three-way ANOVA was used to test the effects of precipitation addition in growing and non-growing seasons and year on soil stoichiometry in the typical steppe of Inner Mongolia. EMMEANS Tukey adjustment were used to test post hoc pairwise comparisons of differences among treatments. A box plot with the same letters are not significantly different from each other at alpha 0.05 probability level. 1 indicates the addition of precipitation, and 0 indicates untreated

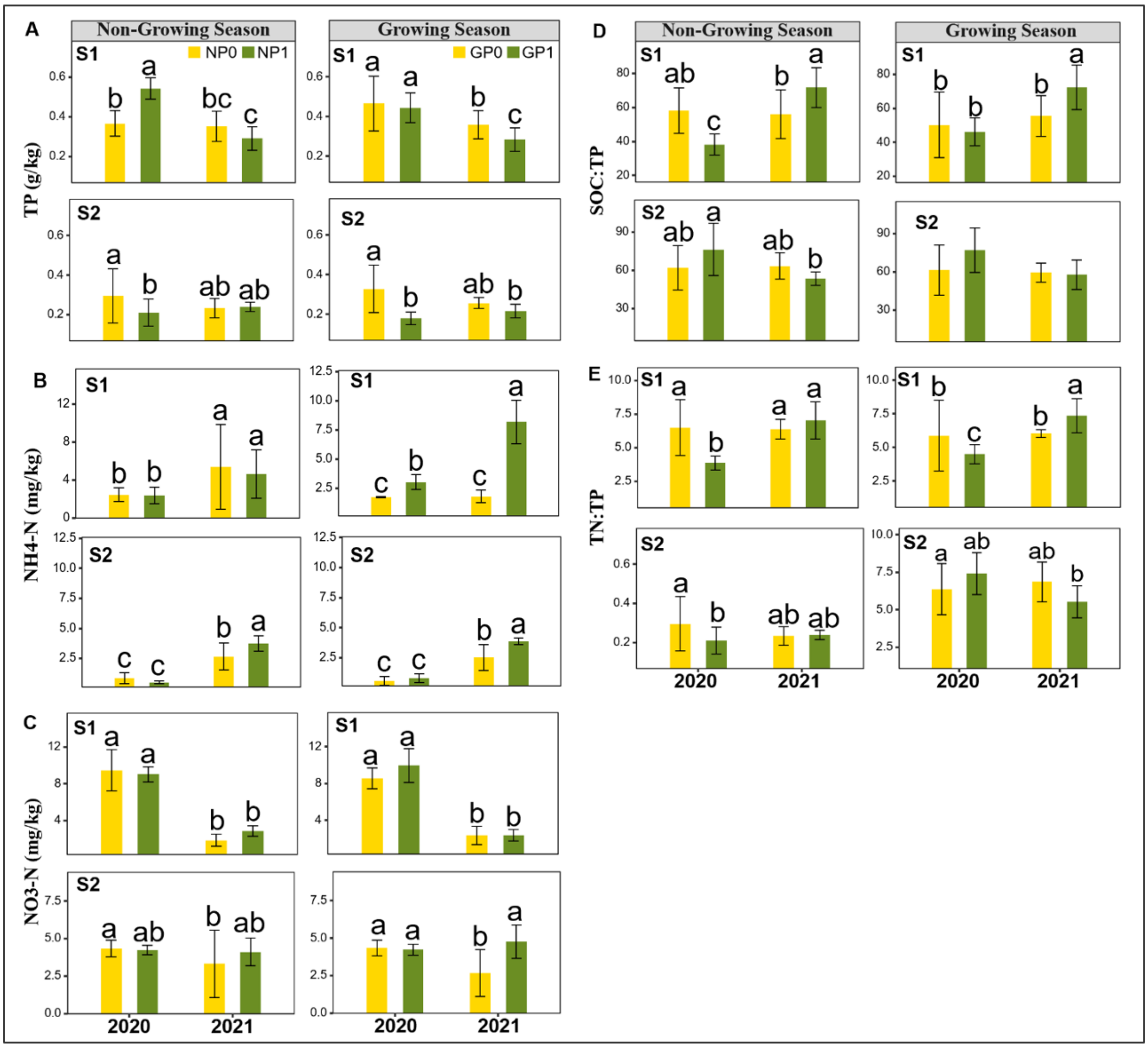

Figure S7 Pearson correlations of community-level aboveground C, N, P, and their ratios with soil nutrients across the 0–20 cm soil depth. Statistical significance is denoted by asterisks: ***: $p < 0.001$; **: $p < 0.01$; *: $p < 0.05$. Aboveground stoichiometry of community level is abbreviated as COMMc, community level carbon, COMMn, community level nitrogen, COMMp, community level phosphorus, and their ratios COMMc:COMMn, COMMc:COMMp, COMMn:COMMp. Similarly, soil nutrients are abbreviated as SOC, Soil organic carbon, TN, Total nitrogen, TP, Total phosphorus, Ava P, Available phosphorus, Ava N, Available nitrogen, NH4-N, Ammonium, NO3-N, nitrate, pH, Soil pH, and their ratios SOC:TN, SOC:TP, TN:TP, and Ava N:Ava P

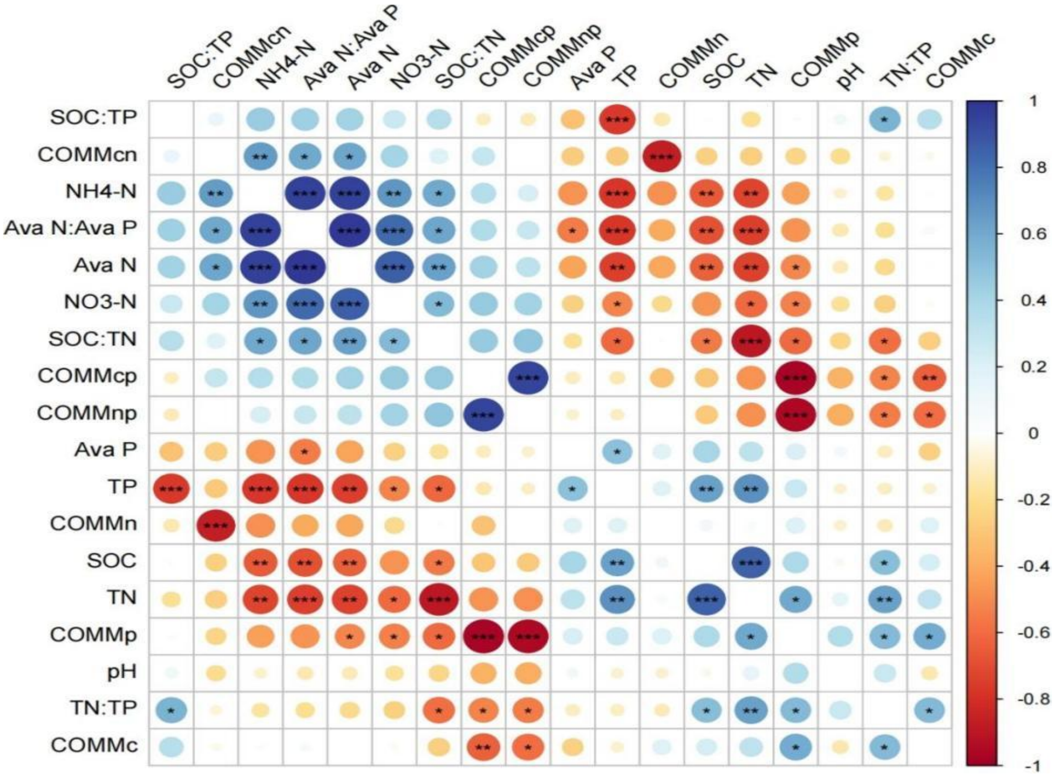

Supplement: Supplementary file 1 [file DataSheet1.pdf]
